# Effects of Poly-Bioactive Compounds on Lipid Profile and Body Weight in a Moderately Hypercholesterolemic Population with Low Cardiovascular Disease Risk: A Multicenter Randomized Trial

**DOI:** 10.1371/journal.pone.0101978

**Published:** 2014-08-01

**Authors:** Rosa Solà, Rosa-M Valls, José Puzo, José-Ramón Calabuig, Angel Brea, Anna Pedret, David Moriña, José Villar, Jesús Millán, Anna Anguera

**Affiliations:** 1 Unitat de Recerca de Lipids i Arteriosclerosi, CIBERDEM, Servei de Medicina Interna, Hospital Universitari de San Joan, IISPV, Facultat de Medicina, Universitat Rovira i Virgili, Reus, Spain; 2 Unidad de Lípidos y Laboratorio de Bioquímica, Hospital Universitario San Jorge, Huesca, Spain; 3 Departamento de Medicina Interna, Hospital Universitario y Politécnico La Fe, Valencia, Spain; 4 Unidad de Lípidos, Servicio de Medicina Interna, Hospital Universitario San Pedro, Logroño, Spain; 5 Centre Tecnològic de Nutrició i Salut, Reus, Spain; 6 Unidad de Hipertensión y Lípidos, Hospital Universitario Virgen del Rocio, Sevilla, Spain; 7 Servicio de Medicina Interna, Hospital Universitario Gregorio Marañón, Madrid, Spain; 8 Medical Department, Rottapharm S.L., Barcelona, Spain; University of Milan, Italy

## Abstract

A dietary supplement (AP, Armolipid Plus) that combines red yeast rice extract, policosanol, berberine, folic acid, coenzyme Q_10_ and asthaxantine can have beneficial effects on cardiovascular disease (CVD) biomarkers. The aim of this study was to assess whether the intake of AP, in combination with dietary recommendations, reduces serum low density lipoprotein cholesterol (LDL-c) concentrations and other CVD biomarkers in patients with hypercholesterolemia. Eligible patients were recruited from the outpatient clinics of six Spanish hospitals *Hospital Virgen del Rocío (Sevilla); Hospital San Jorge (Huesca); Hospital San Pedro (Logroño); Hospital Gregorio Marañón (Madrid), Hospital la Fe (Valencia)* and *Hospital Universitari Sant Joan (Reus)* as recruiting and coordinating center. 102 participants (mean age ± SD; 50.91±11.61; 32 men) with low CVD, with mild-to-moderately elevated LDL-c (between 3.35 mmol/L and 4.88 mmol/L) without hypolipemic therapy were randomized in a double-blind, parallel, controlled, multicenter trial commencing January 2012 and ending December 2012. Among the exclusion criteria were any concomitant chronic disease, triglycerides (TG) >3.97 mmol/L, pregnant or lactating, and history of CVD. At 12 weeks, compared to placebo, AP reduced LDL-c by −6.9%, apolipoprotein (Apo) B-100 by −6.6% and total cholesterol/HDL-c ratio by −5.5%, the ApoB/ApoA1 ratio by −8.6%, while increasing ApoA1 by +2.5% (p<0.05). AP consumption was associated with modest mean weight loss of −0.93 kg (95%CI: -1.74 to -0.12; P = 0.02) compared with control group while dietary composition remained unchanged in the AP group. The AP product was well tolerated. In conclusion, AP, combined with dietary recommendations, reduced LDL-c levels as well as total cholesterol/HDL-c and ApoB/ApoA1 ratios, while increasing Apo A1, all of which are improvements in CVD risk indicators. AP is a product which could benefit patients having moderate hyperlipidemia and excess body weight.

**Trial Registration:**

ClinicalTrials.gov NCT01562080

## Introduction

Cardiovascular diseases (CVD) are the principal cause of death worldwide [1]. Among all the existing risk factors for CVD, atherogenic dyslipidemia is considered preeminent [2]-[6].

Primary prevention of cardiovascular disease by identifying and treating at-risk individuals remains a major public-health priority [2]–[6]. The main pre-emptive approach is a healthy life-style, assisted by pharmaco-therapy according to national and international guidelines [2]-[6]. However, currently available drugs for reducing CVD morbidity and mortality do not have the hoped-for effectiveness in some patients and, as well, the adverse events of the hypolipidemic drugs themselves need to be taken into account [7]. Hence, some patients prefer not to receive drug treatment because they are deeply concerned about possible side effects.

Considerable evidence is available to link several bioactive dietary compounds with the reduction in CVD [8]. A dietary supplement is a product that is orally consumed and which is intended to supplement the usual diet to increase the intake of ingredients reputed to have clinical benefit. These supplements are, usually, an addition to the healthy diet, and not as a conventional food or the sole item of a meal [9].

A dietary supplement (AP, Armolipid Plus, Rottapharm S.L. Barcelona, Spain) has become commercially available and has generated considerable research interest. It combines well-defined, active, natural compounds such as red yeast rice extract, policosanol and berberine (which decrease cholesterol and triglycerides) folic acid (reduces homocysteine) and coenzyme Q_10_ and asthaxantine (anti-oxidants). Recent data support the efficacy of a monacolin K-berberine-policosanol (MBP) mixture as a lipid lowering drug. The MBP mixture was developed and clinically tested after evaluation of the lipid-lowering efficacy of different mixtures of its components: monacolin K and policosanol [10], [11] and berberine and monacolin K [11]. Finally, MBP consumed together with a standard Mediterranean healthy diet appears to be able to reduce low-density lipoprotein cholesterol (LDL)-c and triglyceride (TG) by a mean of 20% in different types of patients [12].

Policosanol is a well-defined mixture of higher aliphatic primary alcohols isolated from sugar cane wax, with cholesterol-lowering effects proven for a dose range from 5-20 mg/day in patients with type II hypercholesterolemia and dyslipidemia associated with non-insulin dependent diabetes mellitus. There is scientific evidence regarding the effect of policosanol as a lipid-lowering agent [13], [14]. Data from *in vitro* studies show that policosanol inhibits hepatic cholesterol synthesis at the mevalonic acid stage of the pathway. Increased hepatic LDL uptake, and increased serum LDL catabolic rates, may also play a role. A systematic review and meta-analysis of randomized controlled studies showed that policosanol was safe, well tolerated and effective for LDL reduction in patients with hyperlipidemia [14]. Controversially, more recent randomized controlled trials do not demonstrate a reduction in lipid levels after policosanol intake in patients with hypercholesterolemia [15], [16].

Red yeast is the product of fermentation of rice by the action of the fungus *Monascus purpureus*. This fungus produces various metabolic byproducts, including a red pigment (hence the name red yeast), bacteriostatic products (i.e. able to inhibit bacterial growth) and an active metabolite, monacolin K, which has been proven to have a lipid lowering effect [17], [18]. The quality of monacolin present in red yeast depends on the strain of *Monascus purpureus* and fermentation conditions. In the case of AP, the red yeast is obtained under standardized conditions of a specific strain of *Monascus purpureus* selected for optimum production of monacolin K, the extent of which is defined and controlled.

Coenzyme Q10 is produced in the body starting from acetyl-CoA in a multi-step process of the mevalonate pathway. The daily 2 mg supplementation of coenzyme Q10 provided by AP has been considered sufficient. The rationale of AP is to combine natural active ingredients with known effects on dyslipidemia at different steps in human metabolism. Further, coenzyme Q10 and astaxanthin are antioxidants that provide cell membranes with protection against free radical and other oxidative attack [19]. The purpose of adding folic acid to AP is to provide the product with a component that is active against hyperhomocysteinemia [20].

Also, berberine, a constituent of AP tablets, has been described as having produced a mild weight loss in obese human subjects [21]. The overall benefit of AP appears to be an improvement in CVD risk in populations with hyperlypidemia and medium-high CVD risk [12], [22]-[25]. The benefits of this dietary supplement have not been studied in a population with early stages of lipid alterations. Thus, the hypothesis is that AP has beneficial effects on CVD biomarkers beyond that of lowering serum LDL-c concentrations. The aims of the present study were to assess whether the addition of AP, in combination with dietary recommendations, decreases the levels of LDL-c and other CVD biomarkers in moderately hypercholesterolemic patients.

## Subjects and Methods

The protocol for this trial and supporting CONSORT checklist are available as supporting information; see Checklist S1 and Protocol S1.

### Design

The study was a multi-centered, randomized, double-blind, placebo-controlled, two-arm study of the effect of AP in individuals with mild-moderate hypercholesterolemia.

The study was approved by the Clinical Research Ethical Committees of all participating centers. Protocols were according to the Helsinki Declaration and good clinical practice guidelines of the International Conference of Harmonization (ICH GCP). This randomized trial was conducted according to extended CONSORT 2010 guidelines. The trial was registered with ClinicalTrials.gov: number NCT01562080. The authors confirm that all ongoing and related trials for this drug/intervention are registered. There have not been any deviations from the study protocol. We declare that there are no restrictions on sharing of data and/or materials. All data and protocols are available from the corresponding author, on request.

### Participants and recruitment

Between January and April 2012, eligible patients were recruited from the outpatient clinics of six hospitals (Hospital Virgen del Rocío, Sevilla; Hospital San Jorge, Huesca; Hospital San Pedro, Logroño; Hospital Gregorio Marañón, Madrid, Hospital la Fe, Valencia) in Spain. The Hospital Universitari Sant Joan de Reus was a recruiting and coordinating center. The trial registration was completed in March 21, 2012 when all six Hospitals confirmed their willingness to participate. Since some administration processes were slower than others, some participating hospitals began recruiting volunteers (n = 118) within the 4-month inclusion period envisaged. The end of study was October 2012.

All the investigators used the same standardized protocols, written manuals, specific guidelines, and materials to train health-care personnel to deliver uniform intervention. The protocol was explained in a training session for all the investigators, and the coordinating center clarified any questions and problems arising in the course of the study follow-up. All participants provided written informed consent prior to participation in the trial.

The participants were community-dwelling men and women >18 years of age, with mild-moderately elevated LDL-c between 3.35 mmol/L [130 mg/dL] and 4.88 mmol/L [189 mg/dL], and were candidates for lifestyle intervention without hypolipemic drug treatment.

Among the exclusion criteria were diabetes mellitus, any concomitant chronic disease, TG) >3.97 mmol/L (350 mg/dL), pregnant or lactating, and a history of CVD.

Participant eligibility or exclusion was assessed by the attending physician and was based on review of clinical records, followed by a screening visit.

### Randomization and intervention

The randomization code was computer generated. Participant assignment to treatment or placebo arm was at a ratio of 1∶1. The number sequence for the subject, center, and treatment assignment were allocated via an interactive electronic response system hosted by the Nutrition and Health Technology Centre (CTNS). The Unit responsible for the randomization took no further part in the study.

After a 1-week baseline lead-in period, screened patients were randomized to receive placebo (microcrystalline-cellulose) or 1 tablet/day of AP (Armolipid Plus, Rottapharm S.L., Barcelona, Spain) as a dietary supplement. The AP tablets contain: red yeast rice extract (200 mg), policosanol (10 mg), berberine (500 mg), folic acid (0.2 mg), coenzyme Q_10_ (2 mg) and asthaxantine (0.5 mg). The trial was for 12 weeks. Blinding was maintained using matching placebo tablets which did not differ from the active AP with respect to appearance or any other physical characteristics.

Treatment monitoring of compliance was with a questionnaire filled-in by patients at clinical interview. Consumption >80% was considered an acceptable level of adherence.

During the intervention, dietary recommendations were disseminated according to the guidelines of the Adult Treatment Panel (ATP) III [5], [6]. At basal and at the end of the clinical visits, dietary compliance was monitored using 3-day dietary records, and confirmed in interviews with the dietician.

The 10-year risk of CHD was calculated using the Framingham Risk Score (FRS) at basal and the end of the outpatient visits. Primary outcome was serum LDL-c concentrations. Secondary outcomes included Total Cholesterol (TC), TG, HDL-c, apolipoprotein (ApoA-1), apolipoprotein B-100 (ApoB-100), glucose, insulin concentrations and HOMA-IR.

At each visit, standard anthropometric data were obtained while participants were wearing lightweight clothing and no shoes. Trained personnel measured weight and height using calibrated scales and well-mounted stadiometer, respectively. Waist circumference (WC) was measured midway between the lowest rib and the iliac crest using an anthropometric tape. All participants were advised to maintain their usual physical activity throughout the study.

### Measurements

A fasting blood sample was taken at 0 and another at week 12. With the subject seated, BP was measured twice with a 1-min interval in between, using an automatic sphygmomanometer. The mean values were used in the statistical analyses.

Screening chemistries and hemogram were performed in each of the participating centers with appropriate clinical chemistry quality controls. All lipids and other biomarkers were measured centrally at the Hospital Universitari Sant Joan, Reus (Catalunya, Spain). Samples were stored at −80°C in the central laboratory's Biobanc (biobanc.reus@iispv.cat) until required for batched analyses. TC, TG, HDL-c, apolipoprotein A-1 (ApoA-1), apolipoprotein B-100 (ApoB-100), and glucose measurements were performed in serum using standard methods on an autoanalyzer (Spinreact, Girona, Spain). LDL-c was calculated by means of the Friedewald formula [26] Insulin was measured in serum using specific ELISA kit (Mercodia AB, Uppsala, Sweden) and the HOMA-IR was calculated [27].

### Statistical analyses

The sample size of 50 subjects in each group was calculated considering a Least Significant Difference (LSD) of 16 mg/dL assuming that the common standard deviation was 28 mg/dL using the Student *t-*test. Setting the bilateral significance level at 5%, the statistical power at 80% and assuming a level of losses of 15%, the total sample required was 116 subjects (58 subjects in each group). We estimated that the minimum statistical difference of 16 mg/dL, represents a sustainable 10% reduction in LDL-c and is a substantial impact on cholesterol management; a reduction that is similar to that of consuming soluble fiber or fitosterols. If the mean LDL-c concentration of the participants was 160 mg/dL the reduction expected will be 16 mg/dL [28].

Descriptive results were expressed as mean ± standard deviation (SD) or percentages, according the type of variable.

The efficacy measured in the continuous variables was analyzed by an ANCOVA (ANalysis of COVAriance) model with baseline value as a covariate. Categorical variables were analyzed using the Fisher exact test. Primary efficacy analysis was conducted on the intention-to-treat (ITT) population, although sensitivity analyses were also performed on the per protocol (PP) and safety populations. Data were analyzed using the SAS software package version 9.2 (SAS Institute Inc., Cary, NC, USA).

## Results

### Characteristics of subjects

**Figure 1:**
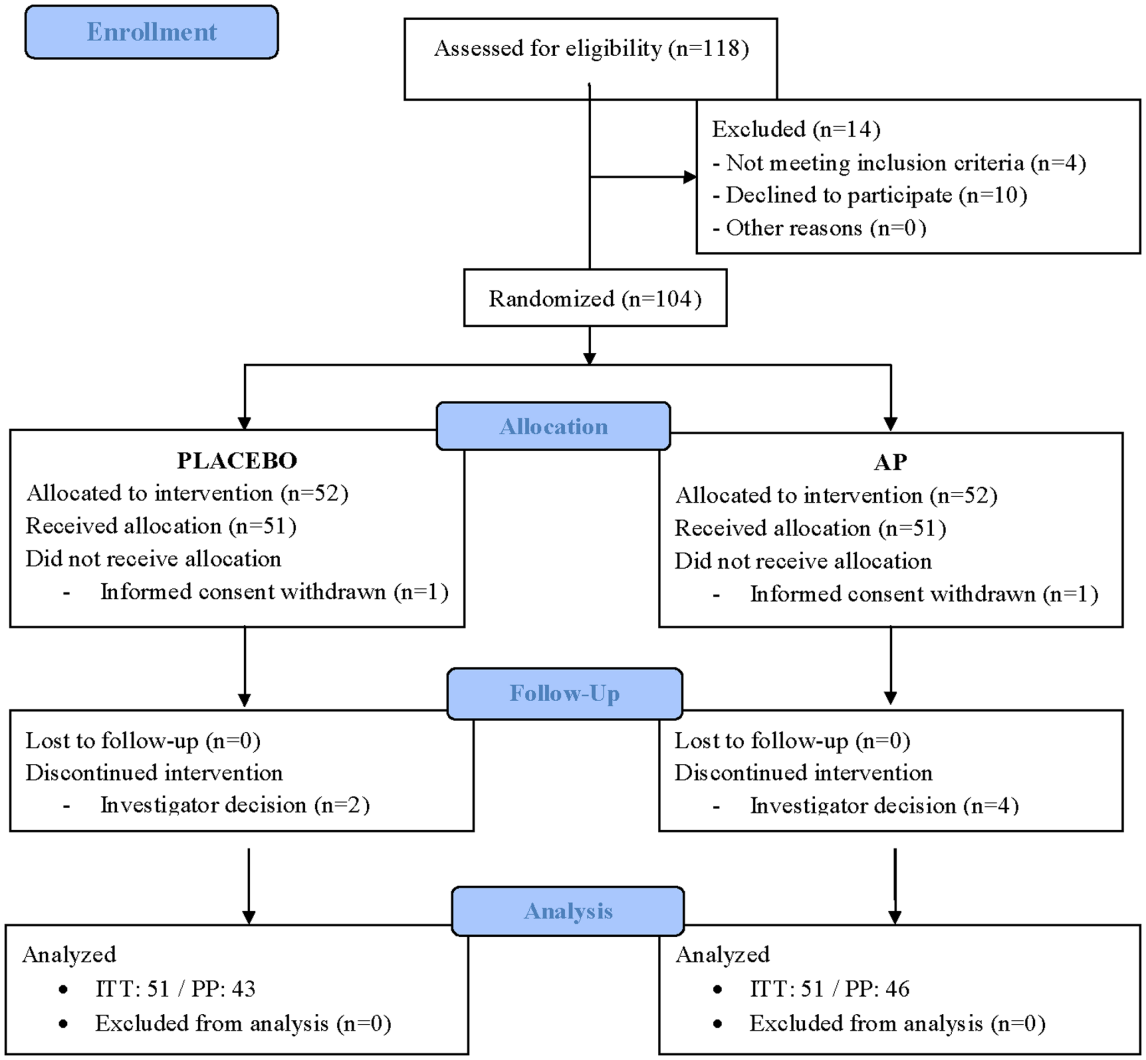
Flow of participants through the study. ITT: intention-to-treat; PP: per protocol, AP: Armolipid Plus.

From the 118 eligible volunteers, 104 were randomized and, finally, data from 102 were analyzed by ITT (Fig. 1). The 14 participants excluded during the screening process were because they did not fulfill all the inclusion criteria, or declined to participate. Two volunteers were excluded after enrolment because informed consent was withdrawn. **Table 1** summarizes the baseline characteristics of study participants. No relevant differences between groups are observed at baseline.

**Table 1:** Baseline characteristics of study participants.

Variable	Intervention^1^ (n = 51)^2^	Placebo (n = 51)
Age; years	49.91±11.61^3^	52.37±11.15
Weight; Kg	68.81±12.62	72.01±15.51
Height; cm	164.68±9.42	161.63±12.96
BMI; Kg/m^2^	25.36±4.07	27.97±8.66
Waist circumference; cm	86.20±11.76	90.40±11.56
Systolic blood pressure; mmHg	122.22±18.14	123.77±17.64
Diastolic blood pressure; mmHg	76.49±12.20	76.75±11.18
Heart rate; bpm	71.43±9.89	70.29±10.09
10-year calculated cardiovascular risk; n (%)		
Low	40 (78.4%)	42 (82.4%)
Medium	7 (13.7%)	8 (15.7%)
High	4 (7.8%)	1 (2.0%)
Smoker; n (%)	16 (31.4%)	7 (13.7%)
Gender; male, n (%)	18 (35.3%)	14 (27.5%)

1 Intervention  =  AP  =  Armolipid Plus; ^2^ Data calculated on intention-to-treat (ITT) population; ^3^ Unless otherwise stated, all results are expressed as mean ± standard deviation (SD).

### Lipid profile

With respect to the main outcomes at 12 weeks, plasma LDL-c in the AP group was reduced by −6.9% compared with placebo. At 12 weeks, relative to placebo, TC was reduced by −4.97% (p<0.05), ApoB-100 was reduced by −6.7% (p<0.05), the ratios of TC/HDL-c by −5.5%, LDL-c/HDL-c by −7.8% and ApoB-100/ApoA-1 by −8.6% (p<0.01). No statistically significant changes were observed in TG and HDL-c levels (**Table 2**). Additionally, we observed that the proportion of subjects achieving LDL-c levels <3.35 mmol/L was similar in the treatment and placebo the groups.

**Table 2:** Lipid profile variables.

Variable		Baseline	Final	Change at 12 weeks relative to baseline	Treatment difference	
		Mean±SD	Mean±SD	Adjusted least square mean [95%CI] (% change from baseline)	Adjusted least square mean [95%CI] (% difference from placebo)	*P*
**Main outcome**						
LDL-c (mg/dL)	Placebo	159.28±15.65^1^	147.55±26.01	−12.78 [−20.06; −4.03] (−8.02%)	−10.46 [−19.81; −1.12] (−6.9%)	0.029
	Intervention^4^	155.67±14.57	135.28±27.15	-23.25 [-27.08; -15.34] (-14.93%)		
**Rest of variables**						
HDL-c (mg/dL)	Placebo	61.10±14.05	60.41±14.49	-2.21 [-5.76; 1.34] (-3.62%)	1.91 [-1.50; 5.32] (3.18%)	0.268
	Intervention	66.51±21.20	67.70±20.34	0.29 [-4.21; 3.62] (0.44%)		
Total cholesterol (mg/dL)	Placebo	243.43±19.49	232.82±28.77	-13.36 [-23.14; -3.58] (-5.49%)	-12.12 [-21.28; -2.95] (-4.97%)	0.010
	Intervention	243.61±24.35	221.81±34.37	-25.48 [-35.98; -14.99] (-10.46%)		
Triglycerides (mg/dL)	Placebo	115.00±56.02	118.86±108.76	5.72 [-25.64; 37.08] (4.97%)	-6.40 [-36.26; 23.47] (-5.6%)	0.671
	Intervention	107.20±61.34	101.15±54.42	-0.68 [-34.45; 33.09] (-0.63%)		
ApoA1 (mg/dL)^2^	Placebo	148.82±11.92	145.76±12.46	-2.82 [-4.88; -0.76] (-1.89%)	3.76 [1.21; 6.31] (2.51%)	0.004
	Intervention	151.88±23.31	152.64±24.96	0.94 [-1.14; 3.03] (0.62%)		
ApoB100 (mg/dL)^3^	Placebo	109.02±16.17	104.37±15.94	-2.20 [-6.36; 1.96] (-2.02%)	-6.87 [-12.07; -1.67] (-6.68%)	0.01
	Intervention	104.31±18.73	94.23±18.53	-9.07 [-13.33; -4.81] (-8.70%)		
Total cholesterol/HDL-c	Placebo	3.99±0.61	4.00±0.67	0.00 [-0.12; 0.12] (0%)	-0.21 [-0.36; -0.06] (-5.5%)	0.007
	Intervention	3.81±0.82	3.60±0.79	-0.21 [-0.33; -0.09] (-5.51%)		
LDL-c/HDL-c	Placebo	2.61±0.45	2.58±0.51	-0.02[-0.13; 0.09] (-0.77%)	-0.19 [-0.33; -0.06] (-7.8%)	0.006
	Intervention	2.44±0.62	2.24±0.64	-0.21 [-0.32; -0.1] (-8.6%)		
ApoB100/ApoA1	Placebo	0.74±0.14	0.72±0.14	0.00 [-0.03; 0.03] (0%)	-0.06 [-0.1; -0.03] (-8.6%)	0.001
	Intervention	0.70±0.17	0.63±0.16	-0.06 [-0.09; -0.03] (-8.57%)		

1 Data calculated on intention-to-treat population (n = 51) and ANCOVA model; ^2^ApoA1: apolipoprotein A1; ^3^ApoB100: apolipoprotein B100; ^4^Intervention  =  AP  =  Armolipid Plus.

### Clinical parameters and glucose and insulin metabolism

At 12 weeks, compared to placebo, the body mass index (BMI) in the AP group was reduced by −5.5% (p<0.05), with a modest mean weight loss of −0.93 kg, (95%CI: −1.74 to −0.12; −1.37%; P = 0.025) while other measured clinical parameters did not change significantly during the intervention. At 12 weeks, compared to placebo, the weight loss observed in the AP consumption group, had no significant impact on LDL-c reduction (P = 0.351), Apo B-100 (P = 0.208), TC/HDL-c ratio (P = 0.565), nor on the ApoB/ApoA1 ratio (P = 0.300). This relationship was analyzed through two complementary approaches. The first was based on an ANCOVA model for LDL-c using the weight-loss as covariate. This resulted in a non-significant contribution of weight-loss (p = 0.351) to LDL-c reduction. The second approach was based on Pearson's correlation coefficient between LDL-c reduction and weight-loss. The result was a small, and non-significant (p = 0.820) contribution. Pearson's correlation coefficient was r = 0.024. In the AP group, energy intake and diet composition were maintained during the study.

There were trends, albeit statistically non-significant, towards reductions in the levels of glucose and insulin, while HOMA-IR was higher in AP group than in placebo group (**Table 3**).

**Table 3:** Clinical variables, glucose concentration and insulin parameters.

Variable		Baseline	Final	Change at 12 weeks relative to baseline	Treatment difference	
		Mean±SD	Mean±SD	Adjusted least square mean [95%CI] (% change from baseline)	Adjusted least square mean [95%CI] (% difference from placebo)	*P*
**Clinical Parameters**						
BMI (Kg/m^2^)	Placebo	27.97±8.66^1^	26.72±3.76	-0.15 [-1.47; 1.16] (-0.54%)	-1.38 [-2.66; -0.10] (-5.5%)	0.034
	Intervention^2^	25.36±4.07	24.61±3.44	-1.53 [-2.96; -0.11] (-6.03%)		
Weight (Kg)	Placebo	72.01 ± 15.51	72.01 ± 15.51	-0.14 [-1.00; 0.71] (-0.19%)	-0.93 [-1.74; -0.12] (-1.37%)	0.025
	Intervention	68.81 ± 12.62	66.92 ± 11.63	-1.08 [-1.99; -0.16] (-1.56%)		
Waist circumference (cm)	Placebo	90.40±11.56	90.39±11.16	0.65[-1.36; 2.66] (0.72%)	0.30 [-1.62; 2.23] (0.39%)	0.754
	Intervention	86.20±11.76	86.24±10.76	0.96 [-1.17; 3.08] (1.11%)		
Systolic blood pressure (mmHg)	Placebo	123.77±17.64	121.25±17.18			
	Intervention	122.22±18.14	122.13±17.62			
Diastolic blood pressure (mmHg)	Placebo	76.75±11.18	75.87±9.96			
	Intervention	76.49±12.20	76.45±10.56			
Heart rate (bpm)	Placebo	70.29±10.09	69.22±9.73			
	Intervention	71.43±9.89	70.38±9.40			
**Glucose and Insulin**						
Glucose (mg/dL)	Placebo	92.77±10.30	92.78±10.50	-1.79 [-5.70; 2.12] (-1.93%)	-1.24 [-4.83; 2.35] (-1.42%)	0.493
	Intervention	90.58±9.27	90.27±10.12	-3.03 [-7.10; 1.03] (-3.35%)		
Insulin (pmol/L)	Placebo	51.75±37.66	55.57±50.69	1.20 [-12.57; 14.97] (2.32%)	-6.53[-23.54; 10.47] (-11.72%)	0.447
	Intervention	56.68±63.68	50.46±35.11	-5.33 [-19.20; 8.55] (-9.40%)		
HOMA-IR	Placebo	1.66±1.26	1.83±1.88	0.1 [-0.40; 0.59] (6.02%)	-0.30 [-0.91; 0.31] (-16.89%)	0.333
	Intervention	1.84±2.56	1.58±1.14	-0.20 [-0.70; 0.30] (-10.87%)		

1 Data calculated on the intention-to-treat population (n = 51). Results from the ANCOVA model; ^2^Intervention  = AP  =  Armolipid Plus.

### Dietary intake

**Table 4** summarizes the dietary intake of study participants. The intake of energy, macronutrients, cholesterol and alcohol did not change during the 12 weeks intervention period, and no significant differences were observed between groups, except towards a higher mono-unsaturated fatty acid (MUFA) percentage and lower fiber content in placebo group relative to the AP intervention group (p<0.05). In the placebo group, the unexpected reduction in fiber consumption did not impact on LDL-c decrease, as evaluated by the Pearson correlation coefficient (r = 0.09, p = 0.408). An ANCOVA model for the changes in LDL-c adjusted for fiber consumption also shows that this effect is non-significant (p = 0.095). In addition, we observed that in the AP group, the fiber consumption is sustained during the study, and no impact on LDL-c concentrations was either expected or observed.

**Table 4:** Composition of participants' diet during the study.

Variable		Baseline	Final	Change at 12 weeks relative to baseline		Treatment difference	
		**Mean±SD**	**Mean±SD**	**Adjusted least square mean [95%CI] (% change from Baseline)**	*P*	**Adjusted least square mean [95%CI] (% difference from placebo)**	*P*
Total energy intake (Kcal)	Placebo	1943.26±926.33^1^	1814.75±595.81	-139.11 [-75.24; 353.46] (-7.16%)	0.200	121.79 [-144.74; 388.32] (8.06%)	0.366
	Intervention^2^	1900.05±603.99	1927.30±776.01	17.15 [-234.35; 200.04] (0.90%)	0.876		
Protein (%)	Placebo	19.03±4.53	18.81±4.44	-0.170 [-0.98; 1.32] (-0.89%)	0.769	-1.149 [-2.86; 0.56] (-0.46%)	0.186
	Intervention	18.02±4.24	17.68±3.96	-0.243 [-0.92; 1.41] (-1.35%)	0.681		
Carbohydrates (%)	Placebo	38.56±7.20	37.66±8.11	-0.862 [-1.68; 3.41] (-2.23%)	0.503	2.143 [1.71; -1.24] (3.02%)	0.212
	Intervention	39.67±7.67	40.18±7.97	0.313 [-2.91; 2.28] (0.79%)	0.811		
Simple carbohydrates (%)	Placebo	19.91±6.27	18.71±5.56	-1.220 [-0.57; 3.01] (-6.13%)	0.178	0.557 [1.24; -1.91] (4.05%)	0.655
	Intervention	19.53±5.88	19.51±6.00	-0.406 [-1.41; 2.22] (-2.08%)	0.658		
Complex carbohydrates (%)	Placebo	18.42±4.82	18.83±6.58	0.488 [-2.29; 1.31] (2.65%)	0.591	1.59 [-1.00; 4.17] (1.42%)	0.226
	Intervention	19.95±6.13	20.56±6.31	0.813 [-2.65; 1.03] (4.07%)	0.383		
Lipids (%)	Placebo	41.47±12.68	41.47±9.20	-0.072 [-3.38; 3.53] (-0.17%)	0.967	-1.93 [-5.45; 1.59] (-1.51%)	0.280
	Intervention	39.96±6.95	39.26±7.74	-0.672 [-2.82; 4.16] (-1.68%)	0.703		
MUFA (%)	Placebo	17.90±3.88	20.14±5.94	2.193 [-3.93; -0.46] (12.25%)	0.014	-1.64 [-3.84; 0.55] (-16.98%)	0.140
	Intervention	19.26±4.38	18.21±4.54	-0.911 [-0.86; 2.68] (-4.73%)	0.310		
PUFA (%)	Placebo	6.91±6.09	5.61±2.67	-1.334 [-0.50; 3.17] (-19.31%)	0.151	0.76 [-1.23; 2.74] (30.56%)	0.452
	Intervention	5.67±2.17	6.34±6.11	0.638 [-2.50; 1.22] (11.25%)	0.498		
SFA (%)	Placebo	10.93±3.00	10.60±2.46	-0.358 [-0.55; 1.26] (-3.27%)	0.434	-0.42 [-1.37; 0.53] (-2.12%)	0.384
	Intervention	10.77±3.15	10.16±2.50	-0.581 [-0.33; 1.49] (-5.39%)	0.209		
Cholesterol (mg)	Placebo	305.73±138.90	320.37±148.63	12.98 [-57.41; 31.46] (4.25%)	0.563	-42.84 [-98.75; 13.07] (1.31%)	0.131
	Intervention	291.85±127.17	277.80±141.24	-16.23 [-29.03; 61.49] (5.56%)	0.478		
Fiber (g)	Placebo	19.30±8.85	16.45±8.05	-2.809 [-0.33; 5.29] (-14.55%)	0.027	3.48 [0.12; 6.84] (12.51%)	0.043
	Intervention	20.26±8.48	20.12±8.85	-0.414 [-2.11; 2.94] (-2.04%)	0.745		
Alcohol (g)	Placebo	7.13±12.93	7.37±14.19	0.350 [-2.97; 2.27] (4.91%)	0.791	0.15 [-4.71; 5.00] (23.36%)	0.952
	Intervention	5.67±8.30	7.57±9.64	1.603 [-4.28; 1.08] (28.27%)	0.238		

1 Data calculated on the intention-to-treat population (n = 51). Mixed Model for Repeated Measures (MMRM) was used to compare differences between intervention and placebo groups;^ 2^Intervention  =  AP  =  Armolipid Plus.

Further, the composition of the recommended 10% saturated fatty acid (SFA) content remained unchanged in both groups in the study.

### Adverse events and product tolerance

There were no statistically significant differences between groups with respect to adverse events reported. The AP test product was well tolerated.

## Discussion

The present study is the first clinical study performed with AP (Armolipid Plus, containing red yeast rice extract, policosanol, berberine, folic acid, coenzyme Q10 and asthaxantine) with a follow-up of dietary recommendations in moderately hypercholesterolemic patients who were candidates for lifestyle intervention. We verified the hypothesis that the intake of AP, in combination with dietary recommendations, not only decreases the levels of LDL-c but also reduces TC/HDL-c, LDL-c/HDL-c, and ApoB-100/ApoA ratios, while serum ApoA-1 concentrations were increased. Another unique finding of the study was that AP consumption was associated with a modest mean weight loss of -0.93 kg, compared with control group. With respect to weight loss, berberine (500 mg), a constituent of the AP tablets, has been described as having produced a mild weight loss (average 5 lb./subject; ≈2.2 Kg) in obese human subjects [23]. When evaluated as an anti-diabetic product, berberine reduced body weight and caused a significant improvement in glucose tolerance without altering food intake in db/db mice [29]. Similarly, berberine reduced body weight and plasma triglycerides and improved insulin action in Wistar rats fed a high-fat diet. Berberine down-regulated the expression of genes involved in lipogenesis and up-regulated those involved in energy expenditure in adipose tissue and muscle. Berberine treatment resulted in increased AMP-activated protein kinase (AMPK) activity in 3T3-L1 adipocytes and L6 myotubes, increased GLUT4 translocation in L6 cells in a phosphatidylinositol 3_kinase–independent manner, and reduced lipid accumulation in 3T3-L1 adipocytes. These findings suggest that berberine displays beneficial effects in the treatment of diabetes and obesity, at least in part, via stimulation of AMPK activity [29]. Other mechanisms proposed, following berberine (200 mg/kg) intake, include decreased degradation of dietary polysaccharides in high-fat-diet (HFD) mice. Berberine significantly reduced the proportions of fecal Firmicutes and Bacteroidetes of total bacteria in HFD mice. In an *ex-vivo* trial, berberine significantly inhibited the growth of gut bacteria under aerobic and anaerobic conditions. In *in vitro* trials, berberine significantly inhibited the growth of Lactobacillus (a classical type of Firmicutes) under anaerobic conditions. Further, berberine significantly increases fasting-induced adipose factor (Fiaf), a key protein negatively regulated by intestinal microbial expression in intestinal and/or visceral adipose tissues. Berberine significantly increases mRNA expressions of AMPK, PGC1a, UCP2, CPT1a, and Hadhb related to mitochondrial energy metabolism, which may be driven by increased Fiaf expression. These results suggest that antimicrobial activity of berberine would result in decreasing the degradation of dietary polysaccharides, lowering potential caloric intake and, subsequently, systemically activating Fiaf protein, together with expression of genes related to mitochondrial energy metabolism in visceral adipose tissues [30]. These mechanisms could be the underlying contributions of AP to the significant weight loss observed.

At 12 weeks, compared to placebo, the loss in body weight observed in the AP consumption group, had no significant impact on reduction of LDL-c, Apo B-100, total cholesterol/HDL-c ratio nor the ApoB/ApoA1 ratio. The energy and the diet composition were maintained during the study in the AP group. As such, the diet recommendations could be said to have not influenced either the body weight or the lipid profile changes detected. Hence, the observed overall improvements in the metabolic parameters could only be accounted-for by the intake of AP.

In the present study, the patients had low baseline serum LDL-c concentrations (155.3 ±14.56 mg/dL) compared to other similar studies assessing the effect of AP: 170.1±1.1, 174.4±21.9, and 172±16 ([12], [31], [32], respectively). Further, our patients had higher baseline serum HDL-c concentrations and lower serum TG concentrations than these other studies. As such, the differences in baseline lipid concentrations could explain, at least in part, the size of the decrease relative to baseline values of our study. For example, our LDL-c baseline concentration was 155.3 mg/dL with a less-marked reduction of 14.9% while the reduction in LDL-c was 25% when its baseline was 174.4 mg/dL [31]. Hence, it is of note that the hypolipidemic impact of AP is related to baseline TC and LDL-c concentrations i.e. greater reductions from higher baseline concentrations.

With respect to TG, we would not expect any important changes because the baseline levels are well within the population reference ranges for these parameters. Again, this highlights the different profiles of those patients included in other studies in which the levels of both parameters were high at the commencement of the study and, as a consequence, both decreased significantly after AP treatment. The lipid profiles of the patients in our study reflect the inclusion criteria i.e. only patients who were candidates for lifestyle intervention were recruited. These individuals have mild-moderate alterations in the lipid profile and, as such, do not meet the ATPIII criteria for treatment with drugs such as statins. The majority of our patients reflected a low, but present, CVD risk at baseline.

Guidelines for lipid management are based, mainly, on LDL-c concentrations [2]-[4]. This is despite other parameters including the TC/HDL-c ratio being better CVD risk predictors than LDL-c alone. The use of these ratios as CVD risk predictors is based on several epidemiological studies which have shown that these indices (TC/HDL-c and LDL-c/HDL-c) are better correlated with CVD and, therefore, are better predictors of CVD than merely lipid concentrations alone [33], [34].The therapeutic goal is to keep the TC/HDL-c ratio below 5∶1 and the optimum ratio is 3.5∶1 [35]. In our study, the AP group showed a TC/HDL-c ratio of 3.6±0.67, which is close to the optimum ratio. Available evidence shows that ApoB-100 and ApoA-1 and, more importantly, the ApoB-100/ApoA-1 ratio could predict cardiovascular heart disease and stroke risk more accurately than conventional lipid measurements; a value <1 is recommended [36]-[38]. In the present study, the lowest mean ApoB-100/ApoA-1 ratio was 0.63 and was observed in the AP consuming group at 12 weeks.

There were trends towards reductions in the levels of glucose, insulin and HOMA-IR, which were greater in the AP group than in placebo, albeit statistical significance was not reached.

The mechanisms underlying the hypolipidemic effect of AP could be based on its main constituents of red yeast rice, policosanols and berberine. As such, AP can inhibit HMG-CoA reductase (the rate-limiting enzyme in the intra-cellular production of cholesterol) which results in a decrease in the endogenous production of cholesterol and an increase in its clearance. The hypocholesterolemic effect observed can be the result of poly-active compounds present in AP. A tablet of AP contains policosanol (10 mg), red yeast rice (200 mg, equivalent to 3 mg of monacolin K), berberine (500 mg), folic acid (0.2 mg), astaxanthin (0.5 mg) and coenzyme Q10 (2 mg). At doses ranging from 10 to 20 mg per day, policosanol lowers TC by 17% to 21% and LDL-c by 21% to 29%, and raises HDL-c by 8% to 15% (13). The weighted estimates of percentage change in LDL-C are -23.7% for policosanol, about 12 mg/day (range 5–40 mg/day. These data are obtained from 1528 patients in 29 eligible studies [13]. Ten mg/day is the dose most studied and it has been shown to be safe and effective. In long-term studies (2 years) with 10 mg/day of policosanol, no significant differences were observed in the tolerability, when compared with placebo [39].

Red yeast rice contains 14 active compounds called monacolins, which inhibit hepatic cholesterol synthesis [40]. Of them, monacolin K is chemically identical to lovastatin [41]. The quality of monacolin present in red yeast depends on the strain of *Monascus purpureus* and fermentation conditions. In the case of AP, the red yeast is obtained under standardized conditions using a specific strain of *Monascus purpureus* selected for optimum production of monacolin K, the extent of which is defined and controlled. This means that all batches of AP have the same amount of monacolin K i.e. 3 mg per tablet. With respect to red yeast rice, one randomized clinical trial performed in patients with coronary artery disease, showed that an extract of red yeast rice reduced recurrent events by 45% [42]. The participants consumed monacolin K in a dose of between 2.5 and 3.2 mg/capsule; similar to the dose contained in each capsule of AP (3 mg of monacolin K)/day. When compared to the placebo group, monacolins-treated patients experienced a more favorable percentage change in TC (-12.45%, 95%CI: -16.19 to -8.71), LDL-c (-21.99%, 95%CI: -26.63 to -17.36), non-HDL-c (-14.67%, 95%CI: -19.22 to -10.11) [7].

A dose of 500 mg/day in mild or moderate mixed hyperlipidemia patients, berberine significantly reduced both LDL-c and TG. When combined with policosanol, red yeast extract, folic acid, astaxanthin and coenzyme Q10 (as in the AP tablet), berberine increased its hypocholesterolemic activity and maintained its triglyceride-lowering and HDL-increasing activity [31]. Coenzyme Q10 is produced, to a large extent in the body commencing from acetyl-CoA and, in a multi-step process, along the mevalonic acid pathway. The daily 2 mg supplementation of coenzyme Q10 provided by AP has been considered appropriate. Further, berberine has been described as up-regulating the LDL receptor independently of sterol regulatory element binding protein. In experimental studies, berberine raised the LDL-R expression through a post-transcriptional mechanism that stabilizes the mRNA. This mechanism of action is distinct from the one of statins [43]. The consequence is a significant reduction in TC and LDL-c. These hypocholesterolemic effects of the components of AP have been well documented, while reduction of Apo B-100 had yet to be demonstrated, until the current data. ApoB-100 is the protein component constituent of the atherogenic very-low-density lipoprotein (VLDL), of intermediate-density lipoprotein (IDL) and of LDL particles, each particle containing one Apo molecule. Hence, plasma Apo B-100 levels reflect the total numbers of atherogenic particles [44]. For example, at 12 weeks, compared to placebo, AP reduced LDL-c by -6.9% and ApoB-100 by -6.6%, almost exactly the same percentage reduction, and reinforces the extent of response to treatment.

The present study reinforces the therapeutic potential of dietary supplements such as AP. In conjunction with a recommended diet, AP affords better control of lipid parameters and, as such, offers a new therapeutic tool for patients with intolerance to certain hypocholesterolemic drugs.

Some limitations need to be taken into account in the interpretation of the results of the study. Patients recruited had low, but present, CVD risk. It is conceivable it will be difficult to reduce even more the low CVD risk.

In conclusion, AP combined with dietary recommendations resulted in: reduced LDL-c levels, total cholesterol/HDL-c and ApoB/ApoA1 ratios, and increased Apo A1; all being improvements in CVD risk. AP is a lipid lowering compound with a moderate weight loss effect. As part of a dietary intervention it could be valuable for moderately hyperlipidemic patients with excess body weight.

## Supporting Information

Checklist S1 **CONSORT 2010 checklist.**
(DOC)

Protocol S1 **Trial Protocol.**
(PDF)
