# Supplementary material for: Effects of Poly-Bioactive Compounds on Lipid Profile and Body Weight in a Moderately Hypercholesterolemic Population with Low Cardiovascular Disease Risk: A Multicenter Randomized Trial
Source: PLoS One. 2014 Aug 1;9(8):e101978. doi: 10.1371/journal.pone.0101978 (PMC4118855; doi:10.1371/journal.pone.0101978)
Supplement: Protocol S1 — Trial Protocol. (PDF) [file pone.0101978.s002.pdf]

EFFECTS OF BIOACTIVE COMBINED COMPOUNDS (ARMOLIPID PLUS ®) ON LIPID PROFILE AND CLINICAL CRITERIA OF METABOLIC SYNDROME IN PATIENTS WITH INCREASED SERUM LEVELS OF LDL-C

Protocol ARM-PLUS-LDL-01

Version: Final Version, date 10 June 2011

PROPIETY OF ROTTAPHARM, S.L.,
CONFIDENTIAL
CAN NOT BE USED DISCLOSED OR PUBLISHED
WITHOUT CONSENT OF ROTTAPHARM, S.L.

ROTTAPHARM | MADAUS

SIGNATURE PAGE

Aproved by:

**INVESTIGADOR COORDINATOR**	
Dra. Rosa Solà<br>Hospital Universitari Sant Joan de Reus<br>Reus	Signature and date: 10 June 2011

**SPONSOR**	
Dra. Anna Anguera<br>Medical Director<br>Rottapharm, S.L.	Signature and date: 10 June 2011

INDEX

1 SCHEDULE 5
2 SUMMARY AND GENERAL INFORMATION 5
3 OBJECTIVES 6
4 METHODOLOGY 7
5 SELECTION OF PATIENTS 7
6 TREATMENT DESCRIPTION 9
6.1 Treatment allocation 9
6.2 Concomitant treatments permited and prohibited 9
6.3 Rules for handling the products under study 9
7 DEVELOPMENT OF THE STUDY AND ANALYSIS OF THE VARIABLES 10
7.1 Principal and secondary efficacy variables 10
7.1.1 Principal efficacy variable 10
7.1.2 Secondary efficacy variables 10
7.1.3 Secondary safety variable 11
7.2 Development of the study 11
7.2.1 Schedule of visits and evaluations 11
7.2.2 Biochemical tests 14
7.2.3 Genomic substudy 14
7.2.4 Dietary record and questionnaire of physical activity 14
7.2.5 Risk cardivovascular factors 15
7.2.6 Ten years cardiovascular risk (as Framingham tables) 15
8 ADVERSE EVENTS 15
8.1 Definitions 15
8.2 Non-serious Adverse Events (NSAEs) 16
8.3 Serious Adverse Events (SAEs) 16
8.4 Imputability criteria 18
9 DISCONTINUATION CRITERIA 19
10 ETHICAL CONSIDERATIONS 19
10.1 General considerations 19
10.2 Data confidentiality 19
10.3 Information patient sheet and informed consent 20
11 PRACTICAL CONSIDERATIONS 20
11.1 Compliance with the protocol 20
11.2 Data filing 21
11.3 File storage 22

12 RATIONALE OF SAMPLE SIZE 23
13 STATISTICAL ANALYSIS 23
13.1 General statistical considerations 23
13.2 Description of protocol deviations and population analysis 23
13.3 Treatment of missing values 24
13.4 Descriptive statistics 24
13.5 Inferential statistics 25
14 BIBLIOGRAPHY 26

1 SCHEDULE

Clinical Evaluations	Screening visit (-7 days)	Baseline visit	Week 4 (±3 days)	Week 7 Phone visit (±3 days)	Week 11 Phone visit (±3 days)	Week 12 (±3 days)
Inclusion/Exclusion Criteria	X	X				
Medical History (family history, smoking habits)	X					
Physical Examination[a]		X	X			X
Therapeutic lifestyle recommendation		X				
Diet diary hand in	X		X			
Diet diary collection		X				X
Diet diary reminding					X	
Randomization		X				
Metabolic syndrome criteria		X				X
10-year CHD risk assess		X				X
Major risk factors		X				
Concomitant Medications	X	X	X	X	X	X
Adverse Events		X	X	X	X	X
Medication supply / prescription		X	X			
Blood Chemistry[b]	X	X				X
Serum Pregnancy Test (Females Only)	X					

[a] Including waist circumference, systolic and diastolic blood pressure, weight, heart rhythm, height

[b] Total cholesterol, LDL-cholesterol, HDL-cholesterol, triglycerides and glucose in fasting conditions.

Baseline and week 12: lipidic profile and glucose in fasting coditions.

2 SUMMARY AND GENERAL INFORMATION

A combination of red yeast rice extract, policosanol, berberine, folic acid and coenzyme Q10 (NUT or Armolipid Plus®, Rottapharm SpA, Monza, Italy) has been recently reported to induce significant improvement in lipid parameters[1,2], in patients with mildly elevated plasma cholesterol.

Given the potential effect of NUT on lipid profile, investigation on the efficacy in the setting of cardiovascular prevention will be important to define its position in prevention programs.

Recently, in a large clinical sample of 1344 patients with moderate cardiovascular risk, combination of NUT with dietary, counselling, for 12 weeks, reduces central obesity, improves lipid profile, decrease diastolic blood pressure, ten-year risk of coronary heart disease using the Framingham Risk Score (FRS), and decreases prevalence of Metabolic Syndrome (MetS)[3].

This is a randomized, double-blind, multicenter, parallel-group, controlled study to assess the effect of Armolipid Plus ® in patients with elevated serum levels of LDL-c. The objective of the study is to demonstrate if, along with dietary recommendations, Armolipid Plus ® can improve the profile of patients with elevated serum LDL-c change acting as therapeutic lifestyle (TLC) according to the definition of the modified Adult Treatment Panel III (ATP III)[4]. Also, MetS, FRS and lipid profile will be followed up.

Following the guidelines of the ATP III, the population of this study will be patients with LDL-C levels ≥ 130 mg / dL (no treatment to reduce LDL-C) where therapeutic lifestyle changes are recommended, but not have LDL-C levels high enough to initiate lipid-lowering therapy. You can also include those who have demonstrated effects or contraindications to lipid-lowering drug therapy.

They follow a treatment Armolipid Plus more dietary recommendations or placebo plus dietary recommendations for 12 weeks.

At the end of this period will be assessed if the LDL-C has decreased by 20%.

3 OBJECTIVES

Primary Trial Objective: To investigate whether addition of Armolipid Plus® decreases 20% the baseline level of LDL-cholesterol.

Other parameters of interest that will be evaluated in order to know the effect of Armolipid Plus® are the following:

- 10-year CHD risk (according to Framingham tables).
- Metabolic syndrome criteria
- Triglycerides and HDL-c

4 METHODOLOGY

This is a randomized, double-blind, multicenter, parallel-group, controlled study to assess the effect of Armolipid Plus ® in patients with elevated serum levels of LDL-c.

The study duration is a screening visit followed by 12 weeks of treatment for each patient.

At the screening visit, the patient will be informed about the study, informed consent will be obtained and reviewed the inclusion criteria are met and none of exclusion.

The patient will take treatment from baseline to final visit, one tablet per day during the meal.

5 SELECTION OF PATIENTS

Patients must comply ALL of the following criteria:

Inclusion criteria:

1. Subject must provide signed and dated informed consent before undergoing any trial related procedure.
2. Subject must be 18 years old or older.
3. Serum LDL-c level ≥130 mg/dl.
4. Patients not requiring lipid-lowering drug therapy according to ATPIII guidelines (LDL-C> 189 mg / dL), or who has demonstrated effects or contraindications to lipid-lowering drug therapy (in this case, treatment should be discontinued 1 month before baseline).

5. Patients must be able to communicate, participate and meet the requirements of the study

Patients who meet ANY of the following exclusion criteria will not be included in the study:

Exclusion criteria:

1. On treatment with LDL-c lowering drug therapy as statins, bile acids sequestrants, nicotinic acid, fibric acids or similar.
2. Any lowering cholesterol NUT with sterols, stanols or similar during the previous 7 days. Start taking new nutraceuticals during the study (from 7 days before baseline) or change the dose of nutraceuticals that are being taken before the start of the study
3. Serum triglycerids level >350 mg/dl.
4. Diagnosed familial hypercholesterolemia.
5. If female, pregnancy or breast-feeding. Female subjects of child-bearing potential must have a negative pregnancy urine test at screening and agree on using a contraceptive method (oral contraceptive, IUD, transdermal contraceptive patch, etc).
6. Participation in a clinical trial within 30 days of randomization or intention to participate in a clinical trial during participation in this study.
7. Known hypersensitivity to Armolipid Plus® or any of its components.
8. Any known pre-existing medical condition that could interfere with the subject’s participation in and completion of the study
9. Any other condition which, in the opinion of a physician-investigator, would make the subject unsuitable for enrollment or could interfere with the subject participating in and completing the study.

10. Subjects who are part of the site personnel directly involved with this study or those who are family members of the investigational study staff.

6 TREATMENT DESCRIPTION

Study products are Armolipid Plus ® or placebo tablets. Its composition is as follows:

Armolipid Plus ®: food supplement composed by substances from natural origin that are berberine, red yeast extract, astaxanthin, policosanols, coenzyme Q10, folic acid and excipients s.c.p.

Placebo: microcrystalline cellulose.

Patients will receive an opaque bottle containing treatment (Armolipid Plus ® or placebo) according to the randomization code assignment.

The treatment dose is one tablet daily during meal.

6.1 Treatment allocation

The allocation to each intervention group will be performed randomly distributing the Armolipid Plus ® group plus dietary recommendations or placebo plus dietary recommendations by using random numbers generated by computer in a 1:1 ratio. Each center will have a list of randomization.
The discontinued patients were not replaced.

6.2 Concomitant treatments permited and prohibited

Antihypertensive treatments are allowed but will remain the same dose during the study as much as possible

6.3 Rules for handling the products under study

The study product to will be stored in a cool, dry place at room temperature. The product selected for each patient consists of a box containing 3 jars x of 30 tablets each, who had been identified with the same number of randomization.

The investigator will deliver product to 4 weeks (1 jar) at baseline and provide the rest of the product until the end of the study, the visit of the Week 4.

7 DEVELOPMENT OF THE STUDY AND ANALYSIS OF THE VARIABLES

In this study will be included a total of 116 patients divided into two groups of 58 patients each according to whether they take Armolipid Plus ® or placebo.

7.1 Principal and secondary efficacy variables

7.1.1 Principal efficacy variable

The primary endpoint of this study is to demonstrate whether Armolipid Plus ® lowers serum LDL-C level of 20% in patients with baseline levels of LDL-C ≥ 130 mg / dL.

7.1.2 Secondary efficacy variables

The secondary efficacy variables are:

1. Proportion of patients achieving LDL-C level <130 mg / dL.
2. Cardiovascular risk reduction to ten years at the end of the study.
3. Proportion of patients meeting the criteria for metabolic syndrome at the end of the study.
4. Increased levels of c-HDL at the end of the study.
5. Decreased levels of triglycerides at the end of the study.

- The analytical variables that will be measured in local laboratories include: lipid profile (total cholesterol, LDL-C, HDL-C and triglycerides) and fasting glucose.
- The metabolic syndrome will be evaluated at the end of the study to assess whether there is a decrease of patients who met this criterion compared with

baseline. Metabolic syndrome is defined as the presence of 3 of the following risk factors:

○ Abdominal obesity	Waist circumference
Men	>102 cm
Women	>88 cm
○ Triglycerides	>150 mg/dL
○ HDL-c	
Men	<40 mg/dL
Women	<50 mg/dL
○ Blood pressure	>130/>85 mm Hg
○ Fasting glucose	>110 mg/dL

7.1.3 Secondary safety variable

Tolerability and safety of the product by the registration of adverse events

7.2 Development of the study

7.2.1 Schedule of visits and evaluations

After signing the informed consent, after an extensive explanation of the study purpose, a screening visit will be made where patients will be evaluated to confirm the inclusion criteria and ruled out the exclusion criteria. Patients included will receive the dietary counseling including dietary recommendations. At baseline, patients will be randomized to the Armolipid Plus ® group plus dietary recommendations or placebo plus dietary recommendations. Patients will be required to maintain their usual physical activity.

The visits are described below and schedule visits taken as reference the baseline visit.

Screening visit:

After signing the informed consent, a screening visit will be conducted to confirm the inclusion and exclusion criteria. Prior relevant medical information will be collected. In addition, concomitant medication being taken by the patient and a blood sample is collected to assess biochemical parameters and rule out pathologies (lipid profile (total cholesterol, LDL-C, HDL-C and triglycerides), glucose, creatinine, TSH, hemogram and liver function tests). In addition, in women of childbearing age, a pregnancy test will be performed on urine.
The 3-day dietary record will be given and will be instructed on its use.

Baseline visit (Day 0):

This visit will take about 7 days after the screening visit.
A blood sample, which will be analyzed in part cool for lipid profile and in part will be frozen for later analysis in a central laboratory (Hospital Universitari Sant Joan de Reus) will be collected. In case of having agreed to participate in the genetic substudy, the sample was obtained and frozen for later analysis.

The inclusion and exclusion criteria were reviewed, a physical examination (waist circumference, systolic and diastolic blood pressure, weight, height and heart rate) criteria for metabolic syndrome will be reviewed, the major cardiovascular risk factors will be performed (as ATP III) and the ten-year cardiovascular risk. Information of concomitant medications it is taking and ask about possible adverse events will be collected.
The 3-day diet record completed by the participant will be collected. It will pass a physical activity questionnaire to assess habitual physical activity. Treatment recommendations for lifestyle change regarding the diet as ATP III guides shall be given.

Week 4:

Physical examination (height is not collected) will be conducted. Other dietary record to fill in during the last week of the study will be given. Information on adverse events and changes in concomitant medication will be collected.

The rest of the product to the patient will be given until the end of the study

Week 7:

Visit phone to collect information on adverse events and changes in concomitant medication.

Week 11:

Visit phone to remind the patient to perform the 3-day dietary record and bring in the visit week 12. Collect information on adverse events and changes in concomitant medication.

Week 12:

A blood sample, which will be analyzed in part cool for lipid profile and in part will be frozen for later analysis in a central laboratory (Hospital Universitari Sant Joan de Reus) will be collected.

Physical examination (height is not collected) will be conducted, and 3-day dietary record filled collected.

The criteria for metabolic syndrome and cardiovascular risk ten-year will review and biochemical tests are repeated.

Information on adverse events and changes in concomitant medication will be collected.

It will fill the physical activity questionnaire to assess it in an objective manner possible significant changes in habitual physical activity

Overall compliance of the study will be assessed by the voluntary.

7.2.2 Biochemical tests

In the local laboratory of each participating center, with fresh sample (3 mL) the lipid profile and fasting glucose of participants will be analyzed, both at baseline and at the visit week 12. Also it will extract 5 mL of blood that will be processed and obtained 1.75 mL of serum that will be frozen at -80 ° C for subsequent analysis in the central laboratory of Hospital Universitari Sant Joan de Reus, as an additional sensitivity analysis.

7.2.3 Genomic substudy

In this study, a genomic sub-study for determining polymorphism of genes predisposing to a resistance in the decrease of levels of LDL-C was performed. The collected samples were analyzed in a central laboratory.

Participating patients signed a specific informed consent for this substudy.

7.2.4 Dietary record and questionnaire of physical activity

The dietary record will collect the information from patient intake for 3 consecutive days that consist of 2 weekdays and 1 holiday. It will be held at the beginning and end of the study to see if there have been changes in the nutritional composition in the diet of the participant.

The Physical Activity Questionnaire, valued at METs, will be performed at the beginning and end of the study to assess if there were changes in physical activity by the participant throughout the study

7.2.5 Risk cardivovascular factors

Cardiovascular risk factors are described in the ATP III guidelines and assessed at baseline. These risks are defined as:

• Smoking

• Hypertension (blood pressure> 140/90 mm Hg or antihypertensive treatment)

• Low level of HDL-C (<40 mg / dL) *

• Family history of premature cardiovascular disease (men, first degree family first 55 years; in women, first degree family first 65 years)

• Age (men> 45 years, women> 55 years)

** If the HDL-c >60 mg/dL, it must be present 3 risk factors.*

7.2.6 Ten years cardiovascular risk (as Framingham tables)

See annex I.

8 ADVERSE EVENTS

8.1 Definitions

An Adverse Event (AE) is any untoward medical event that befalls a clinical research patient or subject treated with a medicinal product, which does not necessarily have a causal relationship with the use of the treatment. An AE is therefore any unfavourable and unintentional sign (including an abnormal laboratory finding), symptom or disease temporarily associated with the use of a medicinal product (research or marketed), whether or not it is regarded as related to the product. An Adverse Event (AE) is any clinically significant disease, sign, symptom or abnormal laboratory finding which has appeared or deteriorated during the course of a clinical trial, regardless of its causal relationship with the investigational drug. The collection of information on non-serious AE should begin following the first administration of an investigational product. Serious AEs should begin to be

collected once the subject's informed consent to participate in the study has been obtained.

AEs may be reported spontaneously or during the interview and physical examination of a subject. All AEs identified should be noted and described in the relevant page of Serious or Non-serious AEs in the CRF. If the diagnosis of the disease or underlying disorder is known, it will be included instead of the individual symptoms.

Subjects who have AEs that lead to the interruption or suspension of treatment with the investigational product, or those who have AEs that are maintained at the end of their participation in the study, should be monitored, as applicable. If possible, the result of any AE leading to permanent suspension or which is present at the end of the study will be reported, particularly if the AE was regarded by the investigator as definitely, probably or possibly related to the investigational product.

8.2 Non-serious Adverse Events (NSAEs)

Any AE not classified as serious, as defined below, must be noted on the non-serious AE page of the CRF. These AEs will be followed up until they are solved or stabilise, and will be reported as SAEs if they become serious.

8.3 Serious Adverse Events (SAEs)

A serious adverse event should be logged on the Serious Adverse Events (SAE) page of the CRF.

A Serious Adverse Event or Serious Adverse event is any untoward medical event which, at any dose:

- Causes death
- Is life-threatening (defined as an event in which the life of the patient or subject were endangered at the time of the event, and does not refer to an event that could have been fatal had it been more serious?)

- Requires or prolongs hospitalization
- Leads to a persistent or significant disability/incapacity
- Is a congenital anomaly or birth defect
- Is an overdose (defined as accidental or intentional intake of any dose of a product that is regarded as excessive and medically important),
- Is a major medical event (defined as a medical event which, while perhaps not immediately life-threatening, may be fatal or lead to hospitalization, but which based on a suitable medical or scientific judgment may compromise the patient or subject or may require intervention [e.g. medical, surgical] to prevent a serious consequence, as defined above. Examples of major medical events will include, although this list is not to be regarded as exhaustive, intensive treatment of allergic bronchospasm, either in the emergency department or at home, as well as blood dyscrasia or convulsions that do not require hospitalization.

Any serious AE which in the opinion of the investigator, is definitely, probably, possibly or not related to the investigational product must be notified by telephone, wherever possible. Nevertheless, all serious AEs, whether or not they are related to the investigational product, should be reported immediately by fax, and the completed SAE report sheet subsequently sent by mail, as recorded in the CRF. Transmission by fax does not replace the obligation to send the SAE report sheet by mail. A courier service may be used instead of fax. A follow-up report will be required if only limited information is initially available.

Contact data:

Víctor Antona Martín

Servicio de Farmacovigilancia

Telf: 932 988 200

Fax: 934 319 885

e-mail: vantona@rottapharm.es

8.4 Imputability criteria

The following imputability criteria are used in this study:

- **Certain** when there is a reasonable causal relationship between the investigational drug and the SAE. The episode improves with the withdrawal of the investigational drug and reappears when the drug is resumed, provided it is clinically viable.
- **Probable** when there is a reasonable causal relationship between the investigational drug and the SAE. The event improves with the withdrawal of the investigational drug. Treatment need not be restarted.
- **Possible** when there is a reasonable causal relationship between the investigational drug and the SAE. There is insufficient information on the withdrawal of the investigational drug or it is not very clear.
- **Improbable** when there is a temporary relationship with the administration of the investigational drug but there is no reasonable causal relationship between the investigational drug and the SAE.
- **Not related** when there is no temporary relationship with the administration of the investigational drug (too early, late or not taken) or there is a reasonable causal relationship between the SAE and another drug, intercurrent disease or circumstance.

The categories of certain, probable and possible are regarded as being related to the investigational drug. The categories of improbable and not related are regarded as not being related to the investigational drug.

The expression “reasonable causal relationship” means that there are facts (evidence) or arguments that suggest a causal relationship. If the information on the SAE is insufficient or contradictory, the event should be regarded as possibly related until further information is available. Since these events may be promptly reported, each investigator should do his utmost to collect all the extra information necessary.

9 DISCONTINUATION CRITERIA

A patient must finalize treatment during the study in the following cases:

• The researcher believes that discontinuing the product is the best choice for the patient.

10 ETHICAL CONSIDERATIONS

10.1 General considerations

This trial will be conducted according to the of Declaration of Helsinki, the Good Clinical Practice guides of the ICH (International Conference of Harmonization), Rottapharm, S.L.'s SOPs.

10.2 Data confidentiality

The investigator will guarantee that all the persons involved observe the confidentiality of any information on the trial subjects.

All the parties involved in a clinical trial will observe the strictest confidentiality to ensure that the personal or family privacy of the subjects participating in it is preserved. Similarly, suitable measures should be taken to avoid the access of unauthorised personnel to the trial data.

The treatment of the personal data of the subjects participating in the trial, particularly with regard to consent, will comply with the provisions of the Constitutional Law 5/1992 of 29 October and European Directive on Data Protection (95/46/EC).

The data obtained in this study will be checked by a monitor appointed by the sponsor and will be used exclusively to draw scientific conclusions. The identity of the patients is confidential and will be known only by the investigator and his collaborators, the auditors and monitors appointed by the sponsor and the inspectors of the competent authorities.

10.3 Information patient sheet and informed consent

The preparation of the Informed Consent Form is the Investigator's responsibility. It must include all the elements required by the ICH, GCP and the applicable regulatory directives, and should comply with Good Clinical Practices and the ethical principles promoted by the Declaration of Helsinki. The consent form should also include a declaration that the sponsor and the health authorities may have direct access to the subjects' records. Before the study begins, the written approval/favourable opinion of the IEC on the Informed Consent Form and any other information provided to the subjects should be obtained.

11 PRACTICAL CONSIDERATIONS

11.1 Compliance with the protocol

The study will be carried out as described in this approved protocol. All revisions should be discussed and revised by the investigator and the Coordinator.

Any amendment to an already authorised clinical trial protocol must be reported to the Independent Ethics Committees (IEC) involved in it. When the amendment is relevant, a prior report by the IEC involved in the trial

The investigator may not implement any deviation or change in the protocol without the prior revision and documented approval/favourable opinion by the IEC regarding any such amendment, except for any changes whose purpose is to remove an immediate risk for the study subjects. Any significant deviation from the protocol should be documented in the case report form.

If a deviation or change in the protocol is implemented before the approval/favourable opinion of the IEC is secured, in order to remove an immediate risk, any such deviation/change shall be submitted, as soon as possible, to:

- the IEC for its revision and/or favourable opinion

The documentation pertaining to the approval signed by the president or the person designated by the latter in the IEC should be sent to the sponsor.

If the revision is an administrative letter, it should be submitted to the IEC.

If an amendment substantially alters the study design or increases the potential risk for the subject: 1) The Informed Consent form should be revised and be resubmitted to the IEC for revision and approval/favourable opinion; 2) The revised form should be used to obtain the consent of the subjects already included in the study if they are affected by the amendment; 3) the new form should be used to obtain the consent of new subjects before recruitment.

11.2 Data filing

The Investigator should prepare and maintain suitable and accurate records designed to log all the observations and other data relevant to the research in each subject treated with the investigational product or included as a control in the trial. The data recorded in the CRFs derived from the original documents should be consistent with these original documents, or in the case of discrepancy an explanation should be provided.

The case report data (CRD) will be in electronic format. Patients should be identified by randomization code.

The confidentiality of any documents that might identify the subjects must be protected, observing their privacy and the rules of confidentiality in accordance with the applicable regulatory requirements.

The investigator will maintain a sheet of signatures to document the signatures and initials of all the people authorised to input or correct data in the CRFs.

11.3 File storage

The sponsor is responsible for the storage of the trial dossier. The investigator will keep the patient identification codes for at least 15 years after the conclusion or interruption of the trial. The clinical records of the patients and other original data will be stored for the maximum period of time allowed by the Hospital, institution or centre where the trial is carried out

The investigator should contact the sponsor before destroying any record associated with the study. The sponsor will notify the Investigator as soon as it is no longer necessary to keep the trial archives.

Should the investigator withdraw from the study (for example, transfer, retirement), the records should be transferred to another person appointed by mutual agreement (for example, another investigator, IEC). Written notification of this transfer should be sent to the sponsor.

12 RATIONALE OF SAMPLE SIZE

It has been estimated a sample size of 50 patients in each group considering a Least Significant Difference (LSD) of 16 mg / dl assuming that the common standard deviation is 28 using a Student t test. Setting the significance level to 5% bilateral, the statistical power of 80% and a loss of 15%, the total sample required is 116 patients (58 patients in each group).

13 STATISTICAL ANALYSIS

13.1 General statistical considerations

Prior to the opening of the randomization codes and closing the database, a Statistical Analysis Plan (SAP) will be conducted, where a detailed statistical analysis process will be included.

Statistical Annexes of the final report of the study will include the statistical analysis plan, the randomization list, the list of all the individual study data and the results of the analysis performed.

13.2 Description of protocol deviations and population analysis

A blind review of data (Data Blind Review), before closing the database to determine the different study populations will be conducted.

In this review, the major and minor protocol deviations and the precise reasons for exclusions of different study populations will be evaluated.

Study populations:

- Selected Population: all subjects selected for the study.
- Randomized Population: All randomized subjects once past the screening phase.
- Safety Population: All randomized subjects who received at least one dose of study product.

- Population by intention to treat (ITT): all randomized subjects who received at least one dose of study product.
- Population per protocol (PP): all randomized subjects who received at least one dose of study product and have efficacy recorded at baseline and at least one of the measures of effectiveness in subsequent visits (for the primary efficacy endpoint) and have no protocol deviations.

The efficacy analysis will be performed on the ITT population and also further assess the primary efficacy population with PP as a measure of sensitivity.

For analysis of safety parameters safety population will be used.

13.3 Treatment of missing values

The BOCF (Baseline Observation Carried Forward) method for the primary endpoint of the study will be implemented.

Do not use any other method for imputing data in any other variable. - In these cases the available data-approximation "Available Data Only (ADO)" will be used.

13.4 Descriptive statistics

The following descriptive indices are detailed in the statistical report by the nature of the variables:

- In continuous variables: Mean, IC95% mean, SD, minimum, P25, median, P75, maximum and N. Per group and globally.
- In categorical variables: % total column in respect, N of each category. Per group and globally.
- In ordinal variables with few categories (less than 10), will be described with two tables: one with descriptive parameters of continuous variables and the other with the parameters of categorical variables. For ordinal variables with >10 categories it will use the same approximation than the continuous variables.

Summary tables for each of the visits of the absolute values and absolute changes from baseline will be prepared.

In open fields or where it be of interest to list all instances of one or more variables, will be described by lists.

13.5 Inferential statistics

The primary efficacy endpoint, absolute difference of the final visit from baseline LDL cholesterol level will be assessed by ANCOVA model with baseline value as covariate. Adjusted means and 95% by bilateral ANCOVA model will be calculated.

Whenever the characteristics of the secondary variables so allow, will also be assessed using the same ANCOVA model with baseline value as covariate

For other efficacy variables, the appropriate hypothesis test will be applied according to its nature: Fisher exact test for categorical variables, the Student t test for continuous variables, Mann-Whitney test for ordinal variables.

All statistical tests proposed will be conducted under a two-sided significance level of 5%.

No interim analyzes are planned.

14 BIBLIOGRAPHY

[1] Cicero AF, Rovati LC, Setnikar I. Eulipidemic effects of berberine administered alone or in combination with other natural cholesterol lowering agents. A single-blind clinical investigation. Arzneimittelforschung; 2007; 57:26–30.

[2] Trimarco B, Benvenuti C, Rozza F, Cimmino CS, Giudice R, Crispo S. Clinical evidence of efficacy of red yeast rice and berberine in a large controlled study versus diet. XV International Symposium on Mediterr J Nutr Metab; Published online Feb 3rd 2010.

[3] Izzo R, de Simone G, Giudice R, Chinali M, Trimarco V, De Luca N, Trimarco B. Effects of nutraceuticals on prevalence of metabolic syndrome and on calculated Framingham Risk Score in individuals with dyslipidemia. J Hypertens. 2010;28(7):1482-7.

[4] Grundy SM, Cleeman JI, Daniels SR, Donato KA, Eckel RH, Franklin BA, et al. Diagnosis and management of the metabolic syndrome: an American Heart Association/National Heart, Lung, and Blood Institute Scientific Statement. Circulation 2005; 112:2735–2752.

ANNEX I: FRAMINGHAM TABLES TO EVALUATE THE 10 YEARS CARDIOVASCULAR RISK

Men

Estimate of 10-Year Risk for Men

(Framingham Point Scores)

Age	Points
20-34	-9
35-39	-4
40-44	0
45-49	3
50-54	6
55-59	8
60-64	10
65-69	11
70-74	12
75-79	13

Total Cholesterol	Points				
	Age 20-39	Age 40-49	Age 50-59	Age 60-69	Age 70-79
<160	0	0	0	0	0
160-199	4	3	2	1	0
200-239	7	5	3	1	0
240-279	9	6	4	2	1
≥280	11	8	5	3	1

	Points				
	Age 20-39	Age 40-49	Age 50-59	Age 60-69	Age 70-79
Nonsmoker	0	0	0	0	0
Smoker	8	5	3	1	1

HDL (mg/dL)	Points
≥60	-1
50-59	0
40-49	1
<40	2

Systolic BP (mmHg)	If Untreated	If Treated
<120	0	0
120-129	0	1
130-139	1	2
140-159	1	2
≥160	2	3

Point Total	10-Year Risk %
<0	< 1
0	1
1	1
2	1
3	1
4	1
5	2
6	2
7	3
8	4
9	5
10	6
11	8
12	10
13	12
14	16
15	20
16	25
≥17	≥ 30

10-Year risk ______%

Women

Estimate of 10-Year Risk for Women

(Framingham Point Scores)

Age	Points
20-34	-7
35-39	-3
40-44	0
45-49	3
50-54	6
55-59	8
60-64	10
65-69	12
70-74	14
75-79	16

Total Cholesterol	Points				
	Age 20-39	Age 40-49	Age 50-59	Age 60-69	Age 70-79
<160	0	0	0	0	0
160-199	4	3	2	1	1
200-239	8	6	4	2	1
240-279	11	8	5	3	2
≥280	13	10	7	4	2

	Points				
	Age 20-39	Age 40-49	Age 50-59	Age 60-69	Age 70-79
Nonsmoker	0	0	0	0	0
Smoker	9	7	4	2	1

HDL (mg/dL)	Points
≥60	-1
50-59	0
40-49	1
<40	2

Systolic BP (mmHg)	If Untreated	If Treated
<120	0	0
120-129	1	3
130-139	2	4
140-159	3	5
≥160	4	6

Point Total	10-Year Risk %
< 9	< 1
9	1
10	1
11	1
12	1
13	2
14	2
15	3
16	4
17	5
18	6
19	8
20	11
21	14
22	17
23	22
24	27
≥25	≥ 30

10-Year risk ______%

U.S. DEPARTMENT OF HEALTH AND HUMAN SERVICES
Public Health Service
National Institutes of Health
National Heart, Lung, and Blood Institute

NIH Publication No. 01-3305
May 2001
